# Effects of sulfur dioxide and particulate matter pollution on hospital admissions for hypertensive cardiovascular disease: A time series analysis

**DOI:** 10.3389/fphys.2023.1124967

**Published:** 2023-02-20

**Authors:** Fatemeh Nouri, Marzieh Taheri, Mahdi Ziaddini, Jamshid Najafian, Katayoun Rabiei, Ali Pourmoghadas, Sheikh Mohammed Shariful Islam, Nizal Sarrafzadegan

**Affiliations:** ^1^ Heart Failure Research Center, Cardiovascular Research Institute, Isfahan University of Medical Sciences, Isfahan, Iran; ^2^ Isfahan Cardiovascular Research Center, Cardiovascular Research Institute, Isfahan University of Medical Sciences, Isfahan, Iran; ^3^ Student Research Committee, Department of Occupational Health, Isfahan University of Medical Sciences, Isfahan, Iran; ^4^ Hypertension Research Center, Cardiovascular Research Institute, Isfahan University of Medical Sciences, Isfahan, Iran; ^5^ Pediatric Cardiovascular Research Center, Cardiovascular Research Institute, Isfahan University of Medical Sciences, Isfahan, Iran; ^6^ Interventional Cardiology Research Center, Cardiovascular Research Institute, Isfahan University of Medical Sciences, Isfahan, Iran; ^7^ Institute for Physical Activity and Nutrition, Deakin University, Melbourne, VIC, Australia

**Keywords:** sulfur dioxide, particulate matter pollution, air pollution, multipollutant model, hypertensive cardiovascular disease, cubic spline, Poisson regression model, negative binomial regression model

## Abstract

**Background and aims:** Air pollution is a major environmental risk factor and the leading cause of disease burden with detrimental effects on cardiovascular systems. Cardiovascular diseases are predisposed by various risk factors, including hypertension, as the most important modifiable risk factor. However, there is a lack of sufficient data concerning the impact of air pollution on hypertension. We sought to study the associations of short-term exposure to Sulfur dioxide (SO_2_) and particulate matter (PM_10_) with the number of daily hospital admissions of hypertensive cardiovascular diseases (HCD).

**Methods:** All hospitalized patients between March 2010 to March 2012 were recruited with the final diagnosis of HCD based on the International Classification of Diseases 10 (codes: I10-I15) from 15 hospitals in Isfahan, one of the most polluted cities in Iran. The 24-hour average concentrations of pollutants were obtained from 4 monitoring stations. In addition to single- and two-pollutant models, we used Negative Binomial and Poisson models with covariates of holidays, dew point, temperature, wind speed, and extracted latent factors of other pollutants controlling for multi-collinearity to examine the risk for hospital admissions for HCD affected by SO_2_ and PM_10_ exposures in the multi-pollutant model.

**Results:** A total of 3132 hospitalized patients (63% female) with a mean (standard deviation) age of 64.96 (13.81) were incorporated in the study. The mean concentrations of SO2 and PM10 were 37.64 μg/m3 and 139.08 μg/m3, respectively. Our findings showed that a significantly increased risk of HCD-induced hospital admission was detected for a 10 μg/m3 increase in the 6-day and 3-day moving average of SO2 and PM_10_ concentrations in the multi-pollutant model with a percent change of 2.11% (95% confidence interval: 0.61 to 3.63%) and 1.19% (0.33 to 2.05%), respectively. This finding was robust in all models and did not vary by gender (for SO_2_ and PM_10_) and season (for SO_2_). However, people aged 35-64 and 18-34 years were vulnerable to SO2 and PM10 exposure-triggered HCD risk, respectively.

**Conclusions:** This study supports the hypothesis of the association between short-term exposure to ambient SO_2_ and PM_10_ and the number of hospital admissions due to HCD.

## 1 Introduction

Cardiovascular diseases (CVDs) have been the leading cause of mortality since 1980 worldwide and predominantly contribute to the burden of disease in the Eastern Mediterranean Region (Mokdad et al., 2018). CVDs, including chronic heart failure, coronary heart disease, and stroke, are predisposed by a variety of risk factors, including hypertension (Roth et al., 2020). Hypertension, as the most important modifiable risk factor (Cohen et al., 2017), not only causes the overall health burden in the community but also severely jeopardizes patients’ quality of life (Trevisol et al., 2011; Riley et al., 2019). An ongoing longitudinal population-based cohort study showed a high incidence of CVD risk factors, especially hypertension, with a 7-year incidence proportion of 22.8% (confidence interval (CI): 20.5–25.1) and an average annual incidence of 3.6% in Isfahan, Iran (Sadeghi et al., 2014). Furthermore, hypertension was the most predominant risk factor for all morbidity and mortality due to CVDs in Isfahan, Iran, indicating the highest attributable risks (Sarrafzadegan et al., 2011; Sadeghi et al., 2014). Various factors, including lifestyle (physical activity, diet, and smoking), genetics, and the environment, are major contributors to hypertension (Leung et al., 2019; Qin et al., 2021; Li et al., 2022a).

Air pollution, a major environmental risk factor, is a heterogeneous mixture of various gaseous (e.g., sulfur dioxide (SO_2_), carbon monoxide (CO), nitrogen dioxide (NO_2_), and ozone (O_3_)) and particulate matter (PM) compounds. According to the Global Burden of Diseases Study (GBD) report in 2019, air pollution, the main cause of disease burden, especially in low-income and middle-income countries, led to about 12% of all death tolls (Murray et al., 2020). In the Prospective Urban Rural Epidemiology study as a multinational, prospective cohort study, the population-attributable fraction for CVDs induced by ambient air pollution was globally estimated at about 13.9%. In addition, the study highlighted that exposure to ambient air pollution is the highest in low- and middle-income countries (Yusuf et al., 2020).

It has been recognized that exposure to air pollution could significantly and rapidly elevate blood pressure, either acute or chronic, thereby raising hypertension and the risk of CVDs development (Brook and Kousha, 2015; Cai et al., 2016; Choi et al., 2019; Qin et al., 2021). In addition to a potential rise in urgent cardiovascular occurrences, blood pressure elevation may also lead to an increase in emergency visits, specifically regarding greater blood pressure levels *per se* or various related symptoms (Szyszkowicz et al., 2012; Brook and Kousha, 2015). Even though air pollution exposure triggered relatively low adverse effects of elevations in blood pressure, it is capable of causing a large population-attributable risk of hypertension due to its pervasive nature (Szyszkowicz et al., 2012; Cai et al., 2016). Therefore, it is essential to identify the effects of air pollution on hospital admission for hypertension, favoring the early prediction and potential prevention of related diseases and mortality in later life. Some previous studies investigated specifically the relationship between air pollutants and hypertension-related hospital admissions. Findings derived from previous studies demonstrated that short-, mid-, and long-term exposure to some gaseous and particulate air pollutants were significantly associated with an increased risk of hospital admissions for hypertension on various lag patterns. However, there is an inconsistency between the effect of some ambient air pollutants and the risk of hypertension (Arbex et al., 2010; Guo et al., 2010; Szyszkowicz et al., 2012; Brook and Kousha, 2015; Chen and Yang, 2018; Tsai et al., 2018; Song et al., 2019; Li et al., 2022a).

Recent systematic reviews and meta-analyses assessing the relationship between ambient air pollution and hypertension highlighted the lack of data concerning some pollutants (such as SO_2_) and the limited number of studies in many low- and middle-income countries, despite being highly polluted regions with prevalent hypertension (Cai et al., 2016; Yang et al., 2018; Qin et al., 2021). Low- and middle-income countries differ in their socioeconomic background and air quality profile from high-income countries, where most evidence examining exposure to air pollutants and hypertension morbidity and mortality is mainly derived (Cai et al., 2016; Yang et al., 2018; Qin et al., 2021). Furthermore, since air quality includes several components and people encounter multiple pollutants in real-life situations, the potentially harmful health effects of air pollutants occur in the context of multi-pollutant; therefore, the impacts of several pollutants should be investigated together. However, most previous studies focused mainly on single- or two-pollutants models assessing air pollution without considering the potential effects of multiple pollutants or any adjustment for multi-collinearity (Tsai et al., 2018; Yang et al., 2018; Qin et al., 2021; Li et al., 2022a).

This study aims to assess the effects of SO_2_ and PM_10_ concentrations on the number of daily hospital admissions for hypertensive cardiovascular diseases (HCD) in a highly polluted city of Iran, Isfahan (Kermani et al., 2018), from March 2010 to March 2012, using a large-scale study database (Rabiei et al., 2017) within a Negative Binomial and Poisson regression models framework using multiple lag structures. We also examined the stability and robustness of the effect estimates after adjusting for other pollutants simultaneously (CO, NO_2_, O_3_, and SO_2_/PM_10_) employing multi-pollutant models. Furthermore, the shape of exposure-response curve relationships between air pollutants and daily hospital admissions for HCD are investigated in the multi-pollutant models. Some stratification analyses were further conducted to explore the associations in vulnerable subgroups.

## 2 Materials and methods

### 2.1 Design

The current study examined the daily number of hospital admissions for HCD concerning SO_2_ and PM_10_ (inhalable particulate matter with an aerodynamic diameter <10 μm) levels among individuals residing in Isfahan from March 2010 to March 2012. This study was a part of a large-scale comprehensive study named the Correlation of Air Pollution with hospitalization And mortality of CardIovascular and RespiraTorY diseases (CAPACITY) on the relationship between hospital visits regarding cardiovascular and pulmonary diseases and air pollutants in Isfahan from 20 March 2010, to 20 March 2012. The CAPACITY study was approved by the Regional Bioethics Committee of the Isfahan University of Medical Science. Details about the CAPACITY study were previously presented (Rabiei et al., 2017; Davoodabadi et al., 2019; Khosravi et al., 2020).

### 2.2 Meteorological data and air pollution

The CAPACITY study used 24- hour meteorological data, including temperature (Fahrenheit scale), wind speed (meters per second), and dew point (in percent) from Isfahan’s Meteorological Organization along with the available satellite data (http://www.nesdis.noaa.gov) for the same study period.

To control the probable confounding effects, the hourly average values of weather conditions were calculated for 24 h and used in the current analysis. On each day of the CAPACITY study, hourly concentrations of air quality indicators, including PM_10_ (milligram per cubic meter: µg/m^3^), SO_2_ (ppb: part per billion), NO_2_ (ppb), CO (ppm: part per million), and O_3_ (ppb), were obtained from 4 local traffic monitoring stations, supervised by Department of Environment (DOE) of Isfahan province. PM_2.5_ levels were recorded only for 10 months in 2011 in one station. After calculating the hourly average of each pollutant concentration from all the stations, the daily average concentrations of considered pollutants were computed. Based on the previous suggestions (Song et al., 2019) about the examination of the possible delayed effects between exposure to air pollutants and the onset of events, performing sensitivity analysis for various lag times, and the uncertainty in the specification of the best lag time, we applied multiple lag time patterns, including 0 to 7 single-day and moving average exposure of various days (such as lag 0–1, lag 0–2, lag 0–3, lag 2–5, lag 0–5, and lag 0–7). In the current analysis, meteorological data and air pollutants were similarly lagged.

### 2.3 Hypertension hospitalization data

Trained experts in the medical record section evaluated patients’ medical records to extract information such as name, sex, father’s name, national identification number, home and work address, date of birth, admission time, discharge date, insurance coverage, final diagnosis (based on the International Classification of Diseases 10 (ICD10) codes), and prescribed medications. Then, they entered the data into the hospital information system (HIS). The treatment deputy of the relevant university regularly supervises data entry. In addition, the Office of Statistics and Information Technology of the Ministry of Health constantly evaluates and monitors the HIS of all hospitals in the country (Rabiei et al., 2017). In the current study, hospitals with coronary care units (CCU), emergency wards for cardiac or respiratory patients, or cardiology, internal medicine, and respiratory diseases wards (where the target patients were likely to be admitted) in Isfahan were identified in coordination with the Deputy of Treatment as a supervisory body for all hospitals. From 24 hospitals in Isfahan, 15 eligible hospitals (Sina, Shariati, Sepahan, Askarieh, Amin, Chamran, Sadoughi, Gharazi, Khanevadeh, Noor, Alzahra, Kashani, Amiralmomenin, Isabne Maryam, and Feiz) met the criteria and were chosen to be considered in this study (Rabiei et al., 2017). We collected private, governmental, social security insurance, university-affiliated, and military hospital records. Therefore, the CAPACITY study includes nearly all of the population admitted for HCD in Isfahan within the study period (Rabiei et al., 2017; Karbakhsh et al., 2022).

Patients referred to the hospital were examined by the on-call doctors and were presented with various differential diagnoses. Then, the doctor in charge would conduct the necessary investigations to determine the definite diagnosis, and then at the time of the patient’s discharge, would record the final diagnosis in the patient’s file in HIS. Therefore, during the hospitalization, other differential diagnoses are checked and ruled out, and the final and relatively definite diagnosis of the disease based on the examination by the specialist doctor was registered in HIS with the ICD10 code. More details on quality control procedures can be retrieved elsewhere (Rabiei et al., 2017). In the CAPACITY study, the elective admissions of database were deleted and only the emergency hospitalized patients were considered. The diagnoses were recorded based on ICD10 codes. In the ICD10 approach, subgroups of HCD included essential (primary) hypertension (I10), hypertensive heart disease (I11), hypertensive renal disease (I12), hypertensive heart and renal disease (I13), and secondary hypertension (I15), which were considered the clinical criteria for inclusion in the study. Total HCD admissions were considered the sum of I10-I15. All non-elective and emergency patients with first HCD events were included in the current study. According to patients’ addresses in hospital records, participants who lived in Isfahan, and were hospitalized either in one of 15 hospitals (university-affiliated, private, social security insurance, military, and governmental) in Isfahan or died due to any hypertensive cardiovascular events from 20 March 2011, to 20 March 2012, were included in the study. In the current study, patients under the age of 18 were excluded because of the few hospital admissions of HCD. More details about data collection and quality control procedures were previously reported (Rabiei et al., 2017).

### 2.4 Statistical analysis

In the current study, the daily hospital admission of HCD, air pollutant concentrations, and meteorological data are linked by date. Data were analyzed using Negative Binomial and Poisson time-series regression models to investigate short-term effect estimates of exposure to air pollutants triggering hospitalized admission due to HCD. The Negative Binomial regression model allows the over-dispersion issue compared to Poisson regression. Concentrations of air pollutants were considered to be the independent variables, while the observed daily HCD hospital admission count was the dependent variable in the mentioned models. The exponent of the estimated coefficient is the relative risk in daily hospital admissions for HCD per 10 µg/m3 increase in SO_2_ and PM_10_ concentrations. Relative risk and percent change ([Relative risk −1] × 100%) and their corresponding 95% CI were computed using Stata (version.15; Stata Corp., College Station, TX, United States). To estimate the effect of SO_2_ (or PM_10_) exposure on HCD hospital admissions, we first conducted a single-pollutant model (without adjustment) using various lag structures. The effect estimates were calculated on 0 to 7 single-day and moving average exposure of various days (such as lag 0–1, lag 0–2, lag 0–3, lag 2–5, lag 0–5, and lag 0–7). Single-pollutant models are considered without any adjustment for meteorological data and other pollutants. To control potential confounding, some time-varying weather-related variables, including dew point, temperature, and wind speed, were included as covariates in the models, namely, the Meteorological models. Additionally, holiday (weekend and public holidays vs. weekday) in the current day (lag 0) as a binary variable, was adjusted in the Meteorological models. Two-pollutant models were conducted to evaluate the stability and robustness of SO_2_/PM_10_ effects in different lag patterns. In two-pollutant models, in addition to weather-condition and holiday variables, we adjusted other pollutants (NO_2_, O_3_, CO, and PM_10_/SO_2_) in a pairwise manner, namely, the Two-pollutant and Meteorological model. Based on all single-pollutant, meteorological, and two-pollutant models for SO_2_ and PM_10_, the lag showing the maximal health effect estimate was selected. According to the chosen lags of SO_2_ and PM_10_, analyses stratified by gender (male and female), age categories (18–34, 35–64, and ≥65 years), and season of admission (warm: spring and summer; and cold: autumn and winter) were conducted in the single-pollutant, meteorological, and multi-pollutant models. The multi-pollutant models were performed by controlling for all pollutants. To remove the influence of multi-collinearity between PM_10_/SO_2_ and other pollutants (NO_2_, O_3_, CO, and SO_2_/PM_10_), the exploratory factor analysis used to extract one or multiple latent variables of pollutants (NO_2_, O_3_, CO, and SO_2_/PM_10_) (Li et al., 2018). Then, these latent variables functioning as latent pollution factors along with weather-condition and holiday variables were included in the models, which were called the multi-pollutant models. To compare effect estimates of subgroups, we calculated a Z value based on the standard normal distribution as follows: $Z=\left(\beta_{1}-\beta_{2}\right)/\sqrt{{SE}_{1}^{2}+{SE}_{2}^{2}}$ , where β1 and β2 were regression coefficients for two groups, and ${SE}_{1}$ and ${SE}_{2}$ were their respective corresponding standard errors. A Bonferroni correction was applied for pairwise comparisons of stratifications with more than two groups. *p*-value <0.05 was deemed statistically significant. The linear relationship between air pollutants and HCD hospital admissions was checked using multiple cubic splines with 2–6 knots (Croxford, 2016) for SO_2_ and PM_10_. To determine the best model fit in terms of the number of knots in the cubic spline, the Akaike information criterion (AIC) was calculated. Then selected cubic spline functions were added into multi-pollutant models in the lag showing the maximal health effect estimate in mentioned models. Afterward, the non-linear association was examined by the Likelihood Ratio test (LRT). Finally, the exposure-response relationship curves between SO_2_ and PM_10_ and hospital admissions due to HCD were plotted. A *p*-value less than 0.05 was considered statistically significant.

## 3 Results

We gathered information on 3,132 hospitalized patients aged 18 years old or above with the first HCD events using the data of the CAPACITY study in Isfahan from March 2010 to March 2012. The descriptive characteristics of participants are presented in Table 1. Approximately 63% of the hospitalized patients were women, more than 43% aged 35–64 years, and about 25% of them were older than 65 years. The mean (standard deviation: SD) age was 64.96 (13.81). In addition, the descriptive statistics for the number of hospital admission by season, gender, and age groups are shown in Table 1. There was a mean (±SD) of 4.29 (±2.42) daily hospital admissions for HCD in Isfahan over the study period. Around 57% of hospitalized visits occurred in cold seasons. The average number of daily hospital admission was higher in women, in patients over 65 years old, and in the cold seasons of the year. Furthermore, the descriptive statistics for daily concentrations of air pollutants and meteorological variables are summarized in Table 1. The mean concentrations of SO_2_ and PM_10_ were 37.64 μg/m^3^ and 139.08 μg/m^3^, respectively, for the period of 730 days. The average temperature, wind speed, and dew point were 60.42°F, 5.19 m/s, and 28.76%, respectively; Table 2 depicts the Spearman correlation coefficient between air pollutants and meteorological data. SO_2_ is negatively correlated with O_3_, CO, temperature, wind speed, and dew point (*p*-value<0.05). PM_10_ is positively correlated with O_3_, CO, and temperature; and negatively correlated with wind speed (*p*-value<0.05).

**TABLE 1:** Descriptive statistics for number of hospital admission, daily concentrations of air pollutants, and daily meteorological variables in Isfahan, Iran during March 2010 to March 2012.

		Number	Percent	Mean	SD	Min	Max	25th percentile	50th percentile	75th percentile
Hypertensive cardiovascular disease										
All ages and sex		3,132	100.0	4.29	2.42	0	14	2.0	4.0	6.0
Age groups	18–34 years	91	2.9	0.12	0.35	0	2	0	0	0
	35–64 years	1,352	43.2	1.85	1.43	0	8	1.0	2.0	3.0
	≥65 years	1,689	25.4	2.31	1.64	0	9	2.0	2.0	3.0
Gender	Female	1978	63.2	2.71	1.78	0	9	1.0	2.5	4.0
	Male	1,154	36.8	1.58	1.41	0	7	0	1.0	2.0
Season	Warm	1,348	43.0	3.62	2.12	0	11	2.0	3.0	5.0
	Cold	1784	57.0	4.98	2.53	0	14	3.0	5.0	6.0
Air pollutants										
PM_10_		730	—	139.08	52.25	36.36	528.33	97.02	149.72	170.21
SO_2_		730	—	37.64	28.46	0	150.0	21.40	28.5	40.73
NO_2_		730	—	40.82	20.57	0.01	128.00	29.63	35.36	50.39
O_3_		730	—	30.81	12.92	3	101.00	22.82	30.92	39.64
CO		730	—	4.53	2.25	0	16	2.69	4.00	6.22
Meteorological data										
Temperature		730	—	60.42	18.50	24.5	92.2	42.1	61.9	77.42
Wind speed		730	—	5.19	2.42	0.5	15.5	3.6	4.9	6.2
Dew point		730	—	28.76	8.85	5.7	48.4	22.4	29.7	35.5

PM_10_: Particulate matter with an aerodynamic diameter <10 μm, SO_2_: sulfur dioxide; NO_2_: nitrogen dioxide; O_3_: ozone; CO: carbon monoxide.

**TABLE 2:** Spearman’s correlation test between air pollution concentrations and meteorological data in Isfahan, Iran during March 2010 to March 2012.

Variables		SO_2_	NO_2_	O_3_	CO	Temperature	Wind speed	Dew point
PM_10_	Correlation coefficient	−0.04	−0.05	0.20	0.54	0.11	−0.07	−0.01
	*p*-value	0.31	0.19	<0.001	<0.001	0.002	0.04	0.72
SO_2_	Correlation coefficient	—	0.05	−0.19	−0.35	−0.34	−0.08	−0.29
	*p*-value	—	0.21	<0.001	<0.001	<0.001	0.02	<0.001
NO_2_	Correlation coefficient	—	—	−0.42	−0.16	0.32	−0.03	0.20
	*p*-value	—	—	<0.001	<0.001	<0.001	0.39	<0.001
O_3_	Correlation coefficient	—	—	—	0.45	0.10	0.09	−0.02
	*p*-value	—	—	—	<0.001	0.009	0.01	0.56
CO	Correlation coefficient	—	—	—	—	0.16	−0.04	0.11
	*p*-value	—	—	—	—	<0.001	0.24	0.002
Temperature	Correlation coefficient	—	—	—	—	—	0.401	0.63
	*p*-value	—	—	—	—	—	<0.001	<0.001
Wind speed	Correlation coefficient	—	—	—	—	—		0.27
	*p*-value	—	—	—	—	—		<0.001

PM_10_: Particulate matter with an aerodynamic diameter <10 μm, SO_2_: sulfur dioxide; NO_2_: nitrogen dioxide; O_3_: ozone; CO: carbon monoxide.

**FIGURE 1:**
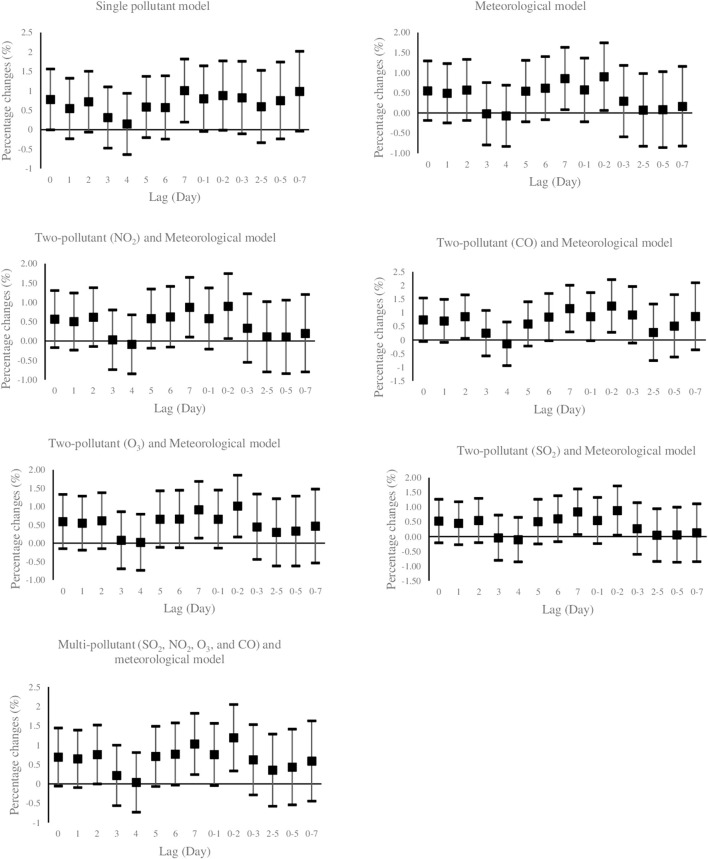
Percent changes (95% CI) of daily hospital admissions for Hypertensive Cardiovascular Disease per 10 μg/m^3^ increase in PM_10_. Lag 0 = current day; Lag 1 = previous 1 day; Lag 2 = previous 2days; Lag 3 = previous 3 days; Lag 4 = previous 4 days; Lag 5 = previous 5 days; Lag 6 = previous 6 days, Lag 7 = previous 7 days, Lag 0–1 = 2-days moving average of lag 0 - lag 1; Lag 0–2 = 3-days moving average of lag 0 - lag 2; Lag 0–3 = 4-days moving average of lag 0 - lag 3; Lag 2–5 = 4-days moving average of lag 2 - lag 5; Lag 0–5 = 6-days moving average of lag 0 - lag 5; Lag 0–7 = 8-days moving average of lag 0 - lag 7. PM_10_: Particulate Matter with an aerodynamic diameter <10 micrometers, SO_2_: sulfur dioxide; NO_2_: nitrogen dioxide; O_3_: ozone; CO: carbon monoxide.

**FIGURE 2:**
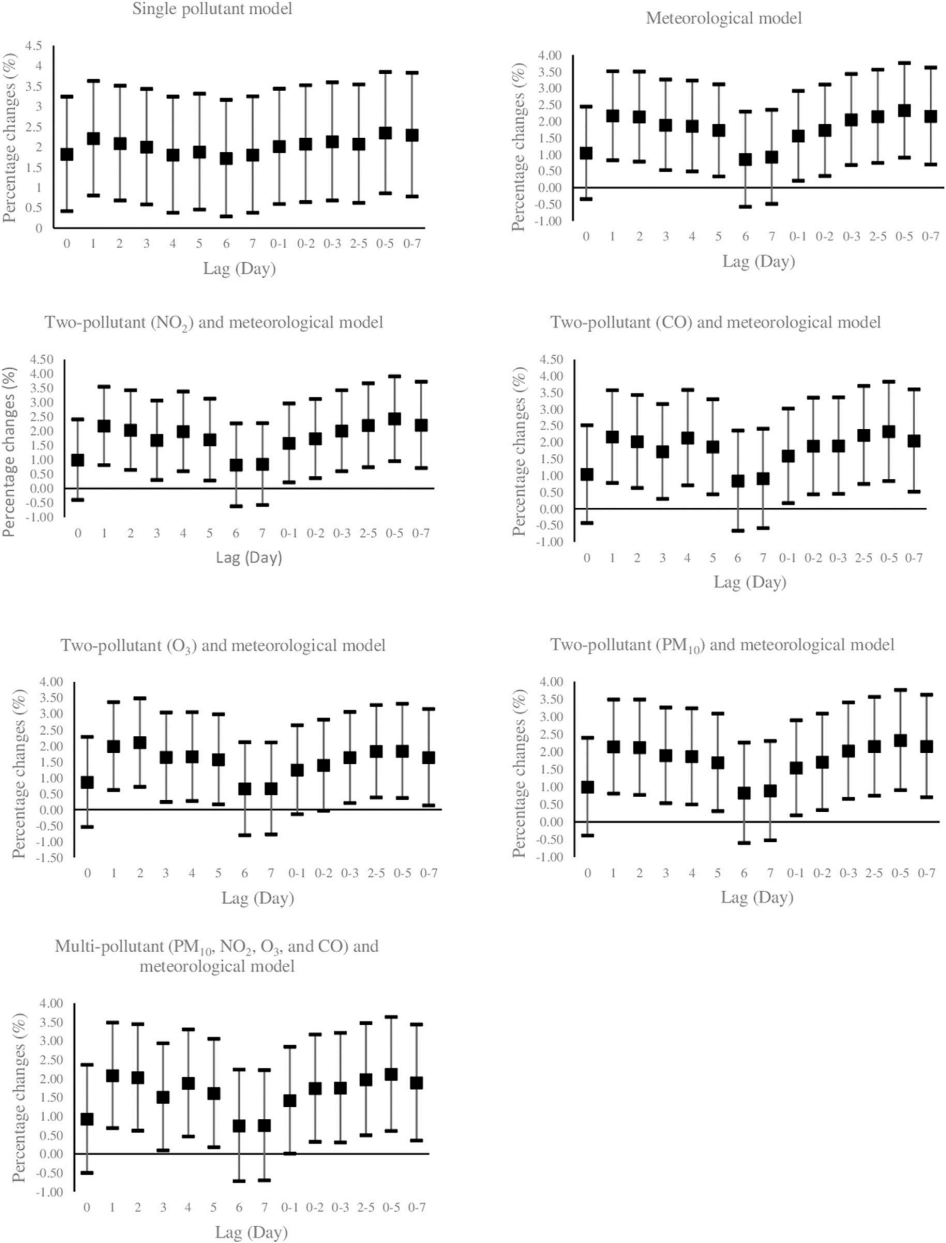
Percent changes (95% CI) of daily hospital admissions for Hypertensive Cardiovascular Disease per 10 μg/m^3^ increase in SO_2_. Lag 0 = current day; Lag 1 = previous 1 day; Lag 2 =previous 2days; Lag 3 = previous 3 days; Lag 4 = previous 4 days; Lag 5 =previous 5 days; Lag 6 =previous 6 days, Lag 7 =previous 7 days, Lag 0–1 = 2-days moving average of lag 0 - lag 1; Lag 0–2 = 3-days moving average of lag 0 - lag 2; Lag 0–3 = 4-days moving average of lag 0 - lag 3; Lag 2–5 = 4-days moving average of lag 2 - lag 5; Lag 0–5 = 6-days moving average of lag 0 - lag 5; Lag 0–7 = 8-days moving average of lag 0 - lag 7. PM_10_: Particulate Matter with an aerodynamic diameter <10 micrometers, SO_2_: sulfur dioxide; NO_2_: nitrogen dioxide; O_3_: ozone; CO: carbon monoxide.

Figure 1 illustrates the effects of PM_10_ exposure (per 10 μg/m^3^ increment) with various lag structures on daily hospital admission for HCD. Data are percentage changes (%) with 95% CIs from single pollutant (without adjustment), meteorological (adjusted for temperature, wind speed, dew point, and holiday), two-pollutant (further adjustment for NO_2_, CO, O_3_, and SO_2_ in a pairwise manner), and multi-pollutant (further adjustment for other pollutants (CO, NO_2_, O_3_, and SO_2_) simultaneously using latent pollution factors by exploratory factor analysis) models. It was found that PM_10_ exposure was positively and significantly associated with the risk of daily hospitalized admissions from HCD at lag 7 (previous 7 days) and lag 0–2 (3-day moving average of lag 0 - lag 2) in the models. The maximal effect estimate was observed on lag 0–2 days. While PM_10_ exposure on several lag days was not significantly related to the daily number of hospital admissions of HCD, the direction of risk on most lags favored positive associations. Furthermore, Figure 2 graphically depicts percent changes (95% CI) in daily hospital admissions for HCD per 10 μg/m^3^ increase in daily SO_2_ concentrations with varying lag patterns using different models. We reached a significant positive relationship between a 10 μg/m^3^ increment in SO_2_ and daily hospital admission for HCD at all lag days except for lag 0, 6, and 7 days in meteorological data and two-pollutant models (after including NO_2_, CO, PM_10_, and O_3_ (additional non-significant lag on 0–1). The maximum effect of SO_2_ appeared at lag 0–5 (6-day moving average of lag 0- lag 5) in all considered models. Therefore, the following analyses only focused on the effect estimates of PM_10_ and SO_2_ on lag 0–2 and 0–5 days, respectively.

We also conducted sensitivity analyses with perfect PM2.5 data (the information derived from one station over the period of 10 months, as previously mentioned). Considering that PM2.5–10 (PM10 minus PM2.5) defines particulate matter between 2.5 and 10 µg/m3 in aerodynamic diameter (Zhang et al., 2016; Shamsipour et al., 2019; Li et al., 2022b), the multi-pollutant models were carried out to estimate the association between SO2 and PM10-2.5 and daily hospital admissions for HCD adjusted for the meteorological variables and simultaneous exposure to other pollutants (NO_2_, O_3_, CO, PM_2.5_, and PM_2.5–10_/SO_2_). The influence of pollutants (SO_2_ and PM_2.5–10_) in both situations (with and without PM_2.5_ adjustment) are almost alike in different lags, as illustrated in Supplementary Figure S1 (in supplementary material); therefore, the findings are stable and robust.

Crude and adjusted relative risks (95% CI) and percent changes (95% CI) for a 10 μg/m^3^ increment in PM_10_ and SO_2_ on the daily number of HCD-related hospital admissions based on gender, age groups (18–34, 35–64, and ≥65 years) and season of admission day (warm and cold) were presented in Table 3. There were no significant relationships between SO_2_ and PM_10_ and the risk of daily hospitalized admission for HCD on warm days in the current study. SO_2_ levels remained significant after the inclusion of meteorological data and latent pollution factors in the single-pollutant model in total and those of 35–64 years old as well as males. In the multi-pollutant models, for a 10 mg/m3 increase in the 6- day moving average of SO_2_ concentrations (lag 0–5) we observed a significant increase of 2.11% (95% CI: 0.61%–3.63%) in the daily hospital admission for HCD. The aforementioned significant associations were 3.14% (95% CI: 1.05%–5.28%) and 3.79% (95% CI: 1.56%–6.07%) in those aged 35–64 years old and males, respectively. However, there was no significant relationship in females, age groups of 18–34 and ≥65 years, and cold seasons of admission days in this study.

**TABLE 3:** Relative risk, percent changes and their 95% confidence intervals in daily hospital admissions from hypertensive cardiovascular disease per 10 μg/m3 increase in PM_10_ and SO_2_.

			Total	Age groups			Gender		Season	
				18–34 years	35–64 years	≥65 years	Female	Male	Warm	Cold
PM_10_	Model 1	Relative risk (95% CI)	1.009 (0.999,1.018)	1.04 (0.99,1.08) ab	1.011 (0.999,1.02) a	1.006 (0.994,1.017) b	**1.012 (1.002,1.02)**	1.003 (0.99,1.015)	0.990 (0.975,1.005)a	**1.014 (1.004,1.024)**a
		*p*-value	0.054	0.09	0.08	0.33	**0.02**	0.66	0.19	**0.003**
		Percent change (95% CI)	0.87 (−0.01,1.77)	3.73 (−0.58,8.23)	1.08 (−0.13,2.31)	0.55 (−0.56,1.68)	**1.21 (0.19,2.24)**	0.28 (−0.97,1.54)	−0.99 (−2.48,0.52)	**1.43 (0.43,2.43)**
	Model 2	Relative risk (95% CI)	**1.009 (1.001,1.017)**	1.045 (0.999,1.09) ab	1.011 (0.999,1.023) a	1.006 (0.995,1.016) b	**1.012 (1.003,1.022)**	1.002 (0.990,1.015)	0.984 (0.967,1.000)a	**1.013 (1.003,1.023)**a
		*p*-value	**0.03**	0.051	0.07	0.27	**0.009**	0.70	0.056	**0.008**
		Percent change (95% CI)	**0.90(0.06,1.74)**	4.49 (−0.02,9.19)	1.09 (−0.09,2.29)	0.59 (−0.45,1.64)	**1.25 (0.31,2.20)**	0.25 (−1.03,1.54)	−1.64 (−3.29,0.04)	**1.33 (0.35,2.32)**
	Model 3	Relative risk (95% CI)	**1.012 (1.003,1.020)**	**1.056 (1.011,1.104)**ab	**1.014 (1.002,1.027)**a	1.008 (0.997,1.018)b	**1.014 (1.005,1.024)**	1.007 (0.994,1.020)	0.990 (0.972,1.008)a	**1.014 (1.004,1.024)**a
		*p*-value	**0.006**	**0.01**	**0.02**	0.16	**0.003**	0.31	0.26	**0.007**
		Percent change (95% CI)	**1.19 (0.33,2.05)**	**5.65 (1.13,10.36)**	**1.44 (0.23,2.66)**	0.76 (−0.31,1.84)	**1.45 (0.48,2.42)**	0.68 (−0.63,2.01)	−1.02 (−2.77,0.75)	**1.36 (0.37,2.37)**
SO_2_	Model 1	Relative risk (95% CI)	**1.023 (1.009,1.039)**	1.032 (0.962,1.108)	**1.031 (1.012,1.051)**a	1.016 (0.998,1.035) a	**1.019 (1.002,1.037)**	**1.030 (1.010,1.051)**	1.006 (0.961,1.053)	1.003 (0.988,1.019)
		*p*-value	**0.002**	0.38	**0.002**	0.09	**0.03**	**0.003**	0.80	0.69
		Percent change (95% CI)	**2.35 (0.86,3.86)**	3.22 (−3.83,10.79)	**3.14 (1.17,5.15)**	1.62 (−0.25,3.52)	**1.93 (0.21,3.67)**	**3.03 (1.001,5.10)**	0.59 (−3.94,5.34)	0.33 (−1.24,1.92)
	Model 2	Relative isk (95% CI)	**1.023 (1.009,1.038)**	1.039 (0.966,1.116)	**1.033 (1.014,1.053)**a	1.013 (0.995,1.032) a	1.014 (0.998,1.031)	**1.038 (1.017,1.059)**	1.007 (0.963,1.053)	1.011 (0.995,1.027)
		*p*-value	**0.001**	0.30	**0.001**	0.15	0.09	**<0.001**	0.75	0.18
		Percent change (95% CI)	**2.32(0.91,3.76)**	3.85 (-3.36,11.60)	**3.33(1.36,5.34)**	1.33 (-0.49,3.18)	1.45 (-0.21,3.13)	**3.75(1.67,5.89)**	0.72 (-3.65,5.29)	1.07 (-0.49,2.66)
	Model 3	Relative risk (95% CI)	**1.021 (1.006,1.036)**	1.012 (0.936,1.094)	**1.031 (1.010,1.053)**a	1.012 (0.993,1.032) a	1.011 (0.993,1.029)	**1.038 (1.016,1.061)**	0.999 (0.951,1.050)	1.009 (0.992,1.026)
		*p*-value	**0.006**	0.77	**0.003**	0.21	0.23	**0.001**	0.99	0.30
		Percent change (95% CI)	**2.11 (0.61,3.63)**	1.18 (−6.41,9.39)	**3.14 (1.05,5.28)**	1.24 (−0.67,3.20)	1.08 (−0.67,2.86)	**3.79 (1.56,6.07)**	−0.04 (−4.87,5.05)	0.88 (−0.78,2.55)

PM10: Particulate Matter with an aerodynamic diameter <10 μm, SO_2_: sulfur dioxide.

Estimates are presented per 10 µg/m3 increase in the lag 0–2 of PM_10_ and lag 0–5 of SO_2_ for age, sex, and season groups. The Negative Binomial time series regression models were used to estimate effects for total, all age and season groups, and females. The poisson time series regression model was used for males.

The statistically significant estimates are highlighted in bold.

Model 1: Single pollutant model (without any adjustment).

Model 2: Adjusted for time-varying meteorological (temperature, wind speed, and dew point) and holiday variables.

Model 3: Multi-pollutant model (further adjustment for latent pollution factors by exploratory factor analysis of carbon monoxide (CO), nitrogen dioxide (NO_2_), ozone (O_3_), and SO_2_/PM_10_).

According to the between-group difference test, each letter (a and b) denotes a subset of groups whose effect estimates differ significantly from each other at the 0.05 level.

According to our findings in Table 3, after controlling for meteorological data and latent pollution factors, data demonstrated significant associations with PM_10_ exposure (lag 0–2) on hospitalized admissions of HCD for total, females, age groups of 18–34 and 35–65 years, and cold seasons. Regarding the multi-pollutant models, the risk of daily hospital admission for HCD affected by PM_10_ exposure was estimated to increase by 1.19% (95% CI: 0.33%–2.05%) in total, 5.65% (95% CI: 1.13%–10.36%) and 1.44% (95% CI: 0.23%–2.66%) in respective age groups of 18–34 and 35–64 years, 1.45% (95% CI: 0.48%–2.42%) in females, and 1.36% (95% CI: 0.37%–2.37%) in cold seasons, respectively. However, there was no association between levels of PM_10_ and risk of daily hospital admission for HCD in males, age group of >65 years, and warm seasons in all single-pollutant, meteorological, and multi-pollutant models.

**FIGURE 3:**
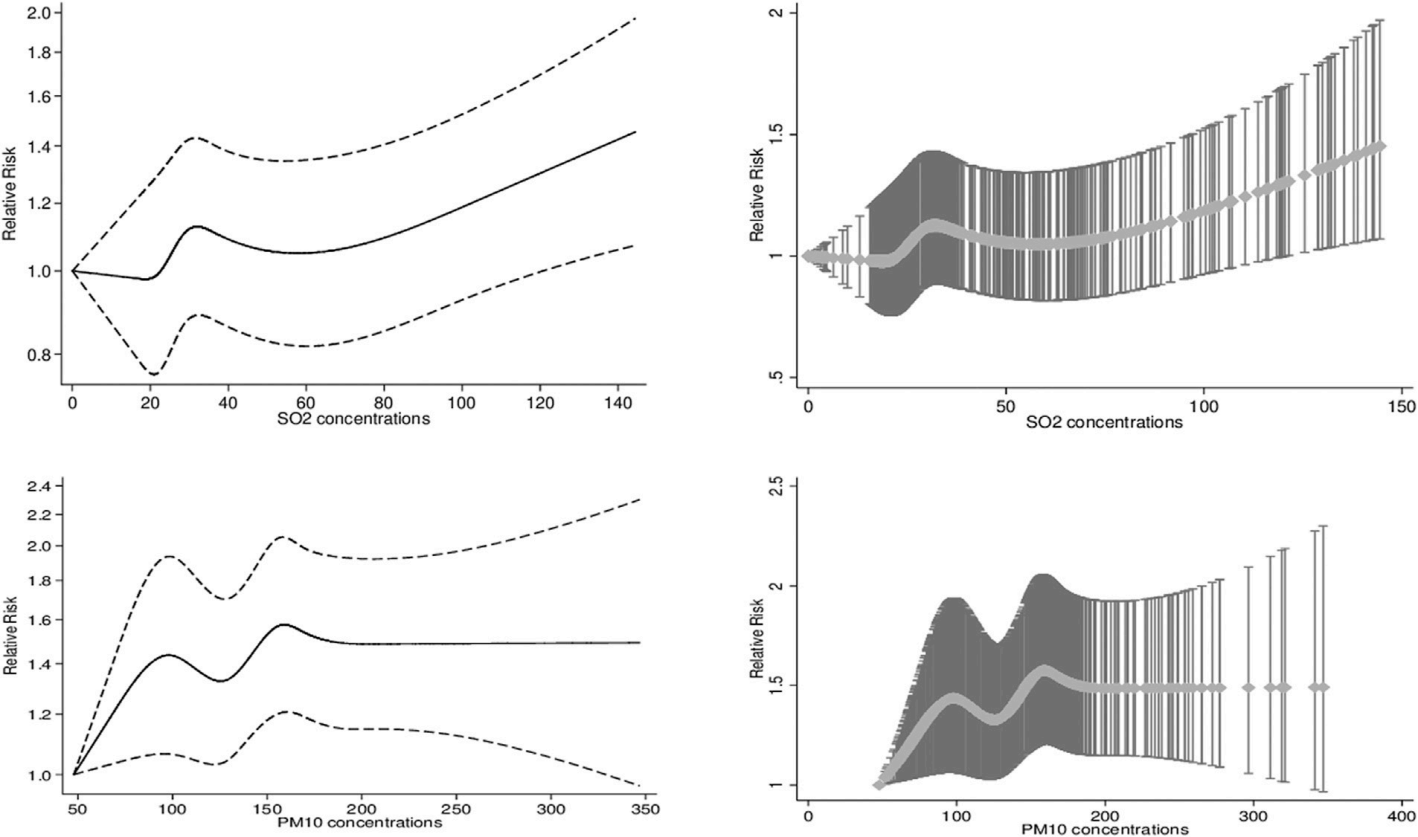
The exposure-response association curves (smoothing by natural cubic spline functions) between levels of Sulfur Dioxide and Particulate Matter 10 and relative risk of hospital admissions for Hypertensive Cardiovascular Disease. The X-axis is the 6-day and 3-day moving average of SO_2_ and PM_10_ concentrations (mg/m3). The Y-axis is the relative risk, after adjusting for holidays, dew point, temperature, wind speed as well as simultaneous exposure to other pollutants (NO_2_, O_3_, CO, and PM_10_/SO_2_).

According to stratified analyses, while the association between SO_2_ exposure and daily hospital admission for HCD did not vary by gender and admission season, age groups of 35–64 and ≥65 years had significant differences in all single-pollutant, meteorological, and multi-pollutant models. It means that people aged 35–64 years were vulnerable to SO_2_ exposure-triggered HCD risk compared with the elderly (65 years old and over). Alternatively, younger people (age group of 18–34 years) were vulnerable to PM_10_ pollution-induced HCD risk compared with those aged 35–64 and >65 years in all models. In addition, the adverse PM_10_ exposure effects led to daily hospital admission for HCD were significantly higher in cold seasons than in warm ones. Furthermore, no effect modifications of gender were found to be significant. Figure 3 shows the exposure-response relationship curve between SO_2_ on lag 0–5 days and PM_10_ on lag 0–2 days and daily hospital admissions for HCD in multi-pollutant models. According to smaller values of the AIC index, cubic spline functions with 4 and 5 knots for SO_2_ and PM_10_ were considered and added into multi-pollutant models, respectively. The exposure-response curve of SO_2_ was S-shaped for concentrations <50 μg/m3 and then tended to become an ascending-shape curve at concentrations ≥50 μg/m3. The curve for PM_10_ showed a steep slope at concentrations <100 μg/m3, then was S-shaped between concentrations >100 and <200, and became flat for ≥200 μg/m3. According to the Likelihood Ratio test, the differences between the linear and spline models for SO_2_ (χ2 = 4.44, *p*-value = 0.21) and PM_10_ (χ2 = 8.57, *p*-value = 0.07) concentrations were not statistically significant.

## 4 Discussion

In the current study, we evaluate the relationship between PM_10_ and SO_2_ concentrations and the daily number of HCD-induced hospital admissions using the data from the CAPACITY study, which is a large-scale study performed in Isfahan, Iran, from March 2010 to March 2012, employing Negative Binomial and Poisson time series regression models. According to the reports of the statistical center of Iran, the population of Isfahan city was nearly 1,756,000 in 2012. Isfahan was one of the most polluted cities in Iran (Kermani et al., 2018). During the period of our study, the mean concentrations of PM_10_ and SO_2_ were 37.64 μg/m^3^ and 139.08 μg/m^3^, respectively, which were higher than recommended standards (Kermani et al., 2018; Karbakhsh et al., 2022). In line with our findings, a previous study on eight Iranian megacities during 2011 and 2012 showed that the second-highest annual average concentration of PM_10_ was in Isfahan, with 127 μg/m^3^, which was approximately 6-times higher than the WHO air quality guideline values (Kermani et al., 2018). The heavy pollution in the cities of Iran is a result of urbanization, industrialization, vehicle accumulation, construction activities, weather condition, dust storms, and population density (Tsiouri et al., 2015; Marzouni et al., 2017; Kermani et al., 2018). Previous studies revealed that the highest impact attributed to PM_10_, with an attributable proportion of 7.037% (5.02–10.27), corresponding to an excess of 637 (435–890) hospital admission of all CVDs (ICD9: 390–459) in Isfahan, during our study period (Kermani et al., 2018). Moreover, recent estimates indicated that in Isfahan, age-, sex-, and place-of-residence adjusted incidence rate (per 10,000) of ischemic heart disease in Isfahan were 41.23–59.92, based on different reference populations during the period between April 2010 to March 2012 (Nouri et al., 2020). In addition, the prospective studies have highlighted a high incidence of various risk factors, including hypertension, obesity, metabolic syndrome, hypercholesterolemia, diabetes mellitus, hypertriglyceridemia, and smoking in Isfahan (Sarrafzadegan et al., 2011; Sadeghi et al., 2014). Given the higher air pollutant concentrations coupled with the ubiquitous nature and the high population at risk in Isfahan, the population-attributable risk induced by air pollution exposure might be remarkably high.

Furthermore, in the current study, a significantly increased risk of HCD-related hospital admission was detected for a 10 μg/m3 increase in SO_2_ (on lag 0–5) and PM_10_ (on lag 0–2) concentrations in an Iranian population with a percent change of 2.11% and 1.19%, respectively, adjusting for meteorological and holidays variables and extracted latent factors of other pollutants in the multi-pollutant model. This finding was robust and did not vary by gender (for both SO_2_ and PM_10_) and admission seasons (just for SO_2_). However, people aged 35–64 and 18–34 were susceptible subgroups to SO_2_ and PM_10_ exposure-induced HCD risk, respectively. Regardless of separate assessments without considering the multi-collinearity of all pollutants in most previous studies, there were some similarities between our results and others on different populations. For example, a study conducted in Ganzhou, China, using a time-series model reported that in single-pollutant models, a 10 μg/m^3^ increase in PM_10_ on the lag day 60 and SO_2_ at lag 62 days was significantly associated with 4.46% (95% CI: 2.86%–5.65%) and 10.99% (95% CI: 5.98%–16.23%) increase in the number of hospitalizations for hypertension (ICD-10: I10–I13), respectively during the years between 2016 and 2020 (Li et al., 2022a). Our findings are in line with the results of this study. Another time-series study conducted by Song et al. in Shijiazhuang, one of the most polluted cities in China, from 2013 to 2016 found that a 10 μg/m^3^ increase of PM_10_ (lag 0–6) corresponded to 0.31% (95% CI: 0.12%–0.50%) increments in the hospitalization of patients for hypertension (ICD_10_ code: I10). Conversely, they observed insignificant associations with SO_2_ on lag 0–4 (0.29%, 95% CI: −0.31%–0.89%). In addition, effect estimates of PM_10_ exposure were more potent in the cold season than in the warm ones, whereas there was no indication of seasonal differences in the associations between levels of SO_2_ and the risk of hospital admissions of patients for hypertension in the aforementioned study. These findings agreed with our current results. In the Song et al. study, the associations of PM_10_ and SO_2_ levels did not vary by sex (likewise to our findings) and age groups of 18–64 and >65 years (in disagreement with ours) (Song et al., 2019)*.* The current observation can be explained by the differences and discrepancies found in the time-activity pattern between old and young subjects (Cohen et al., 2012; Zhang et al., 2016; Yan et al., 2020). Additionally, older individuals exhibit reduced responsiveness to sympathetic and autonomic nervous system stimuli (Esler et al., 1995; Cohen et al., 2012), explaining their declined sensitivity to the hypertensive effects of air pollutants. However, the causal pathway warrants further exploration.

Some previous studies with a case-crossover design, aiming to investigate the association between subjects’ exposure to air pollutants and clinically significant vasopressor responses through emergency department visits for hypertension, demonstrated that various levels of air pollutants (such as SO_2_ and PM_10_) are capable of causing clinically significant health effect related to individual-level blood pressure elevations. They found that recent exposures to air pollutants were capable of raising blood pressure to the extent that induces an emergency department visit for hypertension (Szyszkowicz et al., 2012; Brook and Kousha, 2015). The main findings of a time-stratified case-crossover study showed that a 10 μg/m3 increase in SO_2_ was significantly related to emergency department visits for hypertension (ICD10: I10) at lag 0 days (odds ratio: 1.037 (95% CI: 1.004–1.071), in the single-pollutant model. However, in contrast with ours, after controlling for PM_10_ and NO_2_, the association was no longer statistically significant (Guo et al., 2010). Furthermore, the results of a time-stratified case-crossover study in Taipei, Taiwan, showed that there was no marked association between levels of SO_2_ and the risk of hospital admissions for hypertension (ICD_9_ code 401 equivalent to code I10 based on ICD_10_) from 2009 to 2013 in single- and two-pollutant models, in disagreement with our findings (Chen and Yang, 2018). In addition to original studies, the results of multiple recent meta-analyses of case-crossover, cross-sectional, time-series, cohort, panel studies, or case-control showed that short- and long-term PM_10_ and SO_2_ exposure–hypertension, -systolic, and -diastolic blood pressure association were significant (Cai et al., 2016; Yang et al., 2018; Qin et al., 2021). For example, the results of a meta-analysis concerning long-term effects on adults showed that each 10-μg/m^3^ increase in exposure to PM_10_ and SO_2_ significantly raised the risk of hypertension by 1.04 (Odds Ratio, 95% CI: 1.02–1.07) and 1.21 (Odds Ratio, 95% CI: 1.08–1.36), respectively (Qin et al., 2021). Findings of another meta-analysis demonstrated the relationships between short- and long-term PM_10_ exposures and diastolic blood pressure. There was no significant association with long-exposure to SO_2_. In addition, they found significant associations with short-term exposure to PM_10_ and SO_2_ on hypertension and systolic blood pressure. In contrast with our findings, this study showed a stronger association among men based on stratified analysis (Yang et al., 2018). A retrospective cohort study over 12 years in China revealed that a 10 μg/m3 increase in SO_2_ concentrations was associated with a 76% higher risk of hypertension incidence (hazard ratio: 1.76; 95% CI: 1.163 -1.189) in the fully adjusted model. Based on stratified analyses in this study, the effect estimates of SO_2_ exposure were more pronounced in patients aged 60 or younger, which approximately conforms with our findings (Yan et al., 2020).

In public health assessment, the shape of exposure-response curve relationships is crucial (Yang et al., 2018). In the current study, any noticeable non-linear exposure-response associations between SO_2_ and PM_10_ concentrations and daily hospital admissions for HCD in the multi-pollutant models controlling for holidays, dew point, temperature, wind speed, and simultaneous exposure to other pollutants were not found, which was in agreement with previous time-series studies (Song et al., 2019). Furthermore, in high concentrations of PM_10_, the S-shaped curve tends to a leveling off. It is likely to hospitalization before reasonably high levels of air pollutant concentrations, namely, the harvesting effect (Chen et al., 2017; Song et al., 2019). In contrast with our findings, spline curves illustrated the non-linear relation of SO_2_ (on 3-5 lag hours) with systolic blood pressure from February 2015 to June 2017 in Korea (Choi et al., 2019). The exposure-response curve relations might be varied by meteorological variables, a mixture of air pollutants, geographical variations, and diverse populations with different characteristics (Chen et al., 2017; Yang et al., 2018; Song et al., 2019). Future studies aimed to explore the exposure-response relationship between air pollutant concentrations and hospital admissions may help in the protection of susceptible people.

Further studies in the future would be required to elucidate the precise reasons for the discrepancies between our findings with previous studies. Generally, some of these inconsistencies in related studies might be owing to the consideration of various models, including single-, two-, or multi-pollutant models; controlling for synergic effects (multi-collinearity) of other pollutants in multi-pollutant models; adjusting for several confounders such as meteorological and holiday variables; diverse populations with different geographical and socio-demographic factors; varied lag structures such as single-day or moving average exposure of various days; short-, mid-, or long-term effect estimates; the years under study; different designs, including case-series, case-crossover, and prospective/retrospective cohort study, and thereby different approaches for corresponding statistical models; climate-condition; various proportions of air pollutants; stratified analysis to explore susceptible groups; preexisting non-communicable diseases; various diagnostic criteria for hypertension; and variation in hypertension prevalence (Cai et al., 2016; Yang et al., 2018; Song et al., 2019; Qin et al., 2021).

No accurate mechanism has been proposed yet to determine the association between air pollution and the growing number of hospitalized patients with hypertension. Despite that, recent findings (Sanidas et al., 2017; Vidale and Campana, 2018) suggest that three pathways might attribute to pollution-induced hypertension. First, the classic pathway believes that hypertension results from the provocation of systemic inflammation and oxidative stress. Several inflammatory/pro-oxidative factors can be released into the systemic bloodstream. Second, the alternative pathway, relocating directly into the bloodstream. Some constituents of pollutants might be capable of passing through the alveolar membrane directly into the bloodstream thereby, damaging the vasomotor activity. Third, the central pathway which is the activation of the autonomic nervous system. According to the findings of the previous studies, pollutants can activate receptor-mediated reflexive behaviors in the pulmonary tree and have an impact on the blood pressure, variability, and rhythm of the heart rate. All these mechanisms may have overlapping times and/or occur at different times. Also, the relative importance of the mechanisms would be decided based on the size and type of the pollutants (Sanidas et al., 2017; Vidale and Campana, 2018). More meticulous studies should aim to focus on better clarifying these complex mechanisms in the upcoming years.

### 4.1 Strengths and limitations

Taking limited comprehensive studies from low and middle-income nations, being among immensely polluted countries, and the limited number of published reports about morbidity over mortality data from polluted areas into account (Tsiouri et al., 2015; Kermani et al., 2018; Karbakhsh et al., 2022), our findings add to the limited evidence of the health effects of air pollution in Iran, one of the low-middle-income countries.

The first factor that fortifies the accuracy of the current study is that the CAPACITY included a reliable sample number collected from 15 hospitals across the city incorporating cause-specific morbidity data; so, it was suitable for subgroup analysis. Furthermore, we applied different models, including single-, two, and multi-pollutants models controlling for collinearity of other pollutants with various lag structures such as single- and multi-day lags. Yet, the current study faced some limitations. The restrictions of our study are typical of this type of research. One of the typical concerns in the field of environmental epidemiology is a measurement error of air pollution exposure. To monitor air quality and provide population-average exposure to air pollutants, fixed-site stations were used in the CAPACITY study. Variations in air pollution exposure among people and different places were not fully covered by these stations. Moreover, we were not capable of including different distances of patients from the stations as a covariate in the models. This exposure measurement error leads to an underestimation of the true effect sizes. Furthermore, some factors, including the commuting time and distance, outdoor activity patterns, the time spent outside the home, features of the house, how old the home is, spatiotemporal indicators influencing transmission of air pollutants into the indoor, window-opening pattern and using ventilation and air-conditioning systems, may modify exposure levels of air pollution. This information was not available in the current study. Owing to the lack of monitoring data on PM_2.5_ concentrations in the whole study, this air pollutant was not adjusted for in multi-pollutant models, which might limit our interpretation.

In addition to exposure measurement error, our study includes the impact of measurement error in the outcome variables, which is a typical concern in this field (Szyszkowicz et al., 2012; Chen and Yang, 2018; Song et al., 2019). It is probable that several patients with increased blood pressure as a response to air pollution exposure who had a role in causing acute CVDs might be excluded from this analysis. In the majority of these cases (such as strokes, heart failure, and acute coronary syndromes), visits to the emergency department are classified by type of acute injury of the corresponding organ or cardiovascular events over HCD. Furthermore, information on some factors, such as accurate time of clinical diagnosis and hypertension control measures, was not captured. The level of sensitivity might differ substantially between the current cases compared to new ones (Song et al., 2019). Moreover, data from patients who had other preexisting diseases or health problems (e.g., obesity and diabetes) were not considered in the study due to data limitations. These types of diseases might have a role in declining the patient’s tolerance to pollutants. Although socioeconomic status and educational attainment years, which are related to an individual’s lifestyle and awareness about health protection, affect the health status of patients, these data were not accessible in the current study. Furthermore, it is worth mentioning that using time-series methods can only reveal the relationship between pollutants exposure and the number of hospital admission for HCD, and causality is not proved. The current study assessed cross-sectional data and is not able to investigate the prospective risk for HCD. Finally, this study collected data from only one of the highly polluted cities in Iran, Isfahan, and the generalizability of our results might be circumscribed.

## 5 Conclusion

This study supports the hypothesis of the association between ambient SO_2_ and PM_10_ exposures and the number of hospital admissions of HCD, adjusting for meteorological variables, holidays, and other pollutants simultaneously (NO_2_, CO, O_3_, and PM_10_/SO_2_), along with considering the interdependence (multi-collinearity) of pollutants in the multi-pollutant models. This finding was robust and did not vary by gender (for both SO_2_ and PM_10_) and admission seasons (just for SO_2_). However, people aged 35–64 and 18–34 years were vulnerable to SO_2_ and PM_10_ exposure-triggered HCD risk, respectively. Confirmation of our findings requires further investigations, especially longitudinal studies considering spatial-temporal regression approaches to estimate exact individuals’ exposure to air pollution, as well as overcoming other limitations presented in our study. More accurate quantification of the health effect estimate related to air pollutants exposures would potentially be a necessity for a legislator. Implementation of policies that seek to improve air pollution should continue to be the government’s priority.

## Data Availability

The raw data supporting the conclusion of this article will be made available by the corresponding author on reasonable request, without undue reservation.
