# Supplementary figures and images for: Effects of sulfur dioxide and particulate matter pollution on hospital admissions for hypertensive cardiovascular disease: A time series analysis
Source: Front Physiol. 2023 Feb 20;14:1124967. doi: 10.3389/fphys.2023.1124967 (PMC9986430; doi:10.3389/fphys.2023.1124967)

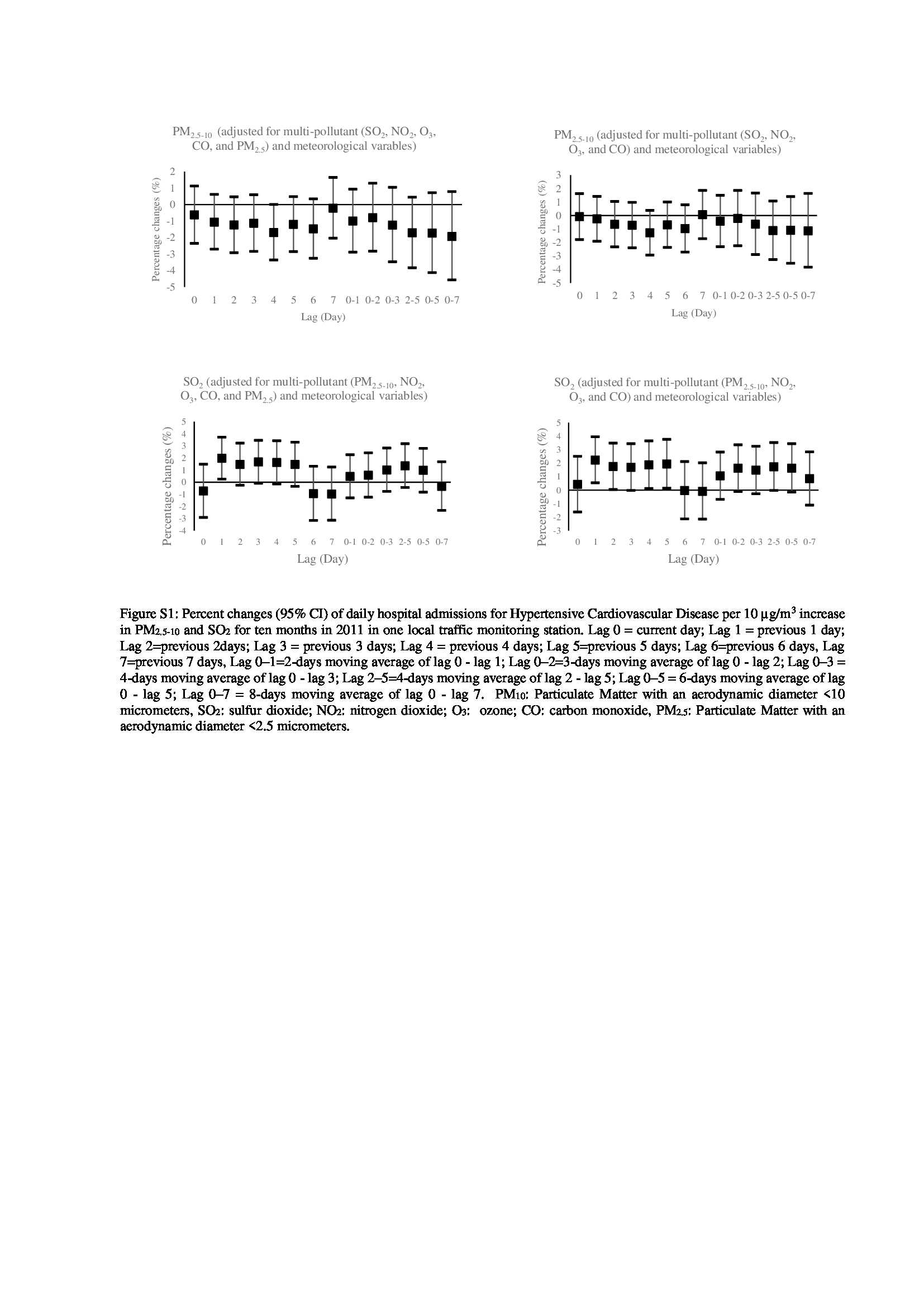

Supplement: Supplementary file 1 [file Image1.jpeg]
